# Supplementary material for: Feasibility of Four Interventions to Improve Treatment Adherence in Migrants Living with HIV in The Netherlands
Source: Diagnostics (Basel). 2020 Nov 20;10(11):980. doi: 10.3390/diagnostics10110980 (PMC7699853; doi:10.3390/diagnostics10110980)
Supplement: Supplementary file 1 [file diagnostics-10-00980-s001.pdf]

diagnostics

Supplementary Table S1. Sociodemographic characteristics of patients participating in the peer support intervention.

	***n* = 23**
**Men (%)**	11 (47.8)
**Receiving cART** [a]	
>6 months	17 (77.3)
<6 months	1 (4.6)
No cART	4 (18.2)
**HIV-RNA >50 copies/ml (%)^**	8 (47.1)
**Mean age (years, sd)**	41.4 (0.51)
**Region of origin (%)**	
Sub Saharan Africa	11 (47.8)
Caribbean and Central America	6 (26.1)
Latin America	5 (21.7)
Other	1 (4.3)
**Heterosexual preference (%)**	17 (73.9)
**Educational attainment (%)**	
No formal education/Primary school	7 (30.4)
Secondary school	7 (30.4)
Higher vocational school	6 (26.1)
University	2 (8.7)
**Employment status (%)**	
Paid employment	4 (17.4)
Unemployed	11 (47.8)
On sick leave	1 (4.3)
Other	7 (30.4)

[a]One matched patient was previously treated with cART due to an acute HIV-infection, *n* = 22. ^All matched patients on cART >6 months at inclusion, *n* = 17.
